# Response shift in hearing related quality of life after cochlear implantation – effect size and clinical significance: a then-test study

**DOI:** 10.1186/s12955-023-02118-w

**Published:** 2023-04-25

**Authors:** Ioana Tereza Brill, Thomas Stark, Lillian Wigers, Stefan Michael Brill

**Affiliations:** 1 Ruhr University Bochum, Bleichstr. 15, 44787 Bochum, Germany; 2 Helios Klinikum München West, Steinerweg 5, 81241 Munich, Germany; 3 Technical University Munich, Arcisstr. 21, 80333 Munich, Germany; 4 Hochschule Kempten, Bahnhofstr. 61, 87435 Kempten, Germany; 5 MED-EL Deutschland GmbH, Moosstr. 7, 82319 Starnberg, Germany

**Keywords:** Cochlear implant, Quality of life, NCIQ, Response shift, Then-test

## Abstract

**Background:**

Quality of life questionnaires are often used in the assessment of rehabilitation of hearing-impaired patients with a cochlear implant. However, a prospective study with a systematic retrospective evaluation of the preoperative quality of life after surgery has not yet been conducted and may reveal a change in internal standards, such as a response shift, due to the implantation and hearing rehabilitation.

**Methods:**

The Nijmegen Cochlear Implant Questionnaire (NCIQ) was used for assessing hearing related quality of life. It has three general domains (physical, psychological and social) and six subdomains. Seventeen patients were tested before (t_0_) and retrospectively (then-test; pre-t_1_) and acutely postoperative (post-t_1_) after cochlear implantation. Observed changes, then-test changes, response shifts and effect sizes were calculated. Non-parametric statistical methods were used.

**Results:**

The NCIQ total score was 52.32 ± 18.69 (mean, standard deviation) for t_0_, 59.29 ± 14.06 for pre-t_1_ and 67.65 ± 26.02 for post-t_1_ questioning. The observed change was statistically significant in all domains but in speech production. Response shift was statistically significant in the total score and in part of the domains. The effect sizes for the response shift were moderate (> 0.5) in the total score, psychological, social general scores and subdomains.

**Conclusions:**

In this study we found that response shift does exist in adults with severe to profound hearing loss undergoing cochlear implantation. By advising the participants to deactivate the implant for the then-test, recall bias and noise were minimized. The clinical significance of the response shift was present in the total score and in the social and psychological domains.

**Trial Registration:**

This study was retrospectively registered with the German Clinical Trial Register, TRN DRKS00029467, on 07/08/2022.

## Background

A cochlear implant (CI) is an electric prosthesis indicated for people with severe to profound hearing loss [1]. Goals of CI provision are: enhance social reconnection; reduce communication effort; enable speech understanding in difficult listening situations (in background noise) [2, 3]; and improve neurocognition in older recipients [4]. Six months of experience with the CI is considered sufficient to achieve an adequately stable state for testing [5, 6].

Quality of life (QoL) assessments are used as an outcome measure in clinical trials [7], including those on CI users [8–10], and is mandatory in the CI register in Germany [11]. Validated health related QoL (HRQoL) questionnaires reliably measure changes [12, 13] and mostly correlate to treatment results. Nevertheless, QoL is a unique, personal perception [14].

In the field of CI, the NCIQ is a well-established, valid and reliable disease-specific questionnaire [8, 15–21]. Most of the benefit in cognitive domains, memory and the quality of life in adult postlingually deafened patients is already achieved at 6 months of CI use [4, 22]. Thus, two time-points for investigation were chosen: preoperative and 6 months after CI-use.

O’Boyle et al. [23] found that adaptability and previous experience can substantially modify perception of QoL. One mechanism in adaptation to changing health is response shift (RS) [24, 25]. Schwartz et al. [26] defined the RS as the change in the meaning of one’s self-evaluation of a target construct. The RS is beneficial but may complicate the correct evaluation of quality of life [27]. RS comprises the change in meaning of QoL over time based on new information acquired [28], so the retrospective judgment is more valid [29]. This change comes from the adjustment of internal standards (recalibration), alteration in the importance attributed to the domains of QoL (reprioritization), and in the definition of QoL (reconceptualization) [24].

One RS method is the then-test method. This is a pre-post treatment questioning to look for a RS and it can be used to estimate recalibration [30].

RS has been investigated in organizational change [31]; education [25]; and various medical fields, such as cancer [27], chronic illness [32], or moderate hearing loss [33], but never in deafness and cochlear implantation.

Hinderink et al. [15], Sanchez-Cuadrado et al. [19, 20], and Ottaviani et al. [21] compared acute preoperative NCIQ results of CI candidates with then-test results of experienced CI users, i.e. results from different groups of subjects. Hinderink et al. [15] inferred a strong agreement between answers from the retrospectively and the acutely questioned groups regarding preoperative QoL. However, from their published data it can be concluded that basic and advanced sound perception scores from the two groups were significantly different. Based on Hinderink et al. [15], Sanchez-Cuadrado et al. [19, 20] and Ottaviani et al. [21] validated Spanish and Italian language versions of the NCIQ, using a similar method of two distinct subject groups. Olze et al. [8] questioned experienced elderly CI patients acutely regarding their current QoL and retrospectively regarding their preoperative QoL, i.e. a single group was questioned twice. They discussed their participants’ pre-CI state data as possibly contaminated by recall bias, while referencing Hinderink et al. [15] with their inferred strong agreement between results.

Considering this state of the literature and because within-subject RS was never studied in CI, we found it important to investigate whether it exists also in this group of patients. This could provide valuable information for preoperative counseling, improve realistic expectations, and help in cost-effectiveness analyses.

The null hypothesis of this study was that participants’ preoperative acute scores and their retrospective then-test scores do not differ, i.e. no RS exists. To our knowledge, this is the first study investigating whether there is a RS in assessing CI users’ QoL.

## Methods

### Study design

This prospective longitudinal monocentric study comprised a retrospective component. It was approved by the ethics committee of the Technical University of Munich (272/20 S). The CI rehabilitation timeline within our clinic is: diagnostics, surgery, device activation, basic and advanced system fitting, speech therapy, medical care for at least one year, and aftercare once a year thereafter. All participants gave their informed written and verbal consent prior to their inclusion in the study.

### Participants

Based on data from Olze et al. [8], we performed an a-priori power calculation for the required number of subjects, for a matched pairs Wilcoxon signed-rank test and normal distribution. Assuming means of 35 and 50, and standard deviations of 20 for both pre- and post-operative acute data, which all are conservative values given the data from Olze, a correlation of 0.7, a required alpha of 0.5 and a power (1-beta) of 0.9, we arrived at a required total sample size of fourteen.

We recruited nineteen postlingually deafened adults implanted between 2018 and 2019 who had also completed acute preoperative NCIQ. Two CI users refused to complete the then-test, arguing, that they had benefited from a hearing aid before the operation and they therefore would not be able to recall the preoperative state well enough. They were not included in the study group. Seventeen participants (*n* = 17) completed all study tasks.

### Testing

All seventeen participants answered the NCIQ before (time t_0_) and after (time t_1_) the surgical procedure. At time t_1_, i.e., after implantation, activation of the device, and sufficient hearing experience of at least six months of use, both now (post-t_1_) and then-tests (pre-t_1_) were completed. The preoperative acute testing at t_0_ took place in the clinic shortly before implantation by filling out the NCIQ forms on paper. The retrospective and acute testing at t_1_ was sent by mail due to the COVID-19 pandemic. For pre-t_1_ testing (then-test), participants were asked to temporarily deactivate their CI and thus restore the state from before the implantation to better recall the earlier situation. Users can deactivate their CI at any time and usually do so daily, if only for sleep.

We used the then-test method for detecting RS, because we consider our group of patients well-suited to overcome one potential limitation of this method, namely the sensitivity to recall bias, by temporarily deactivating the implant. This could lead to more reliably assessing the RS effect size. Besides other clinical studies, most relevantly to our study, Joore et al. [33] in the field of hearing aids also employed the then-test, which renders our results suited for comparison. Other established methods to detect RS are based on various applications of regression analysis, which are not suited well for our relatively small group of participants.

### Measures

The NCIQ is a disease-specific HRQoL questionnaire with 60 items, subdivided into three general domains and six subdomains [15]. The physical general domain comprises the subdomains “basic sound perception” (BSP), “advanced sound perception” (ASP), and “speech production” (SP). The psychological general domain is congruent with the subdomain “self-esteem” (SE) and the social general domain includes the subdomains “activity level” (AL) and “social interaction” (SI). Each subdomain consists of ten items. The answers are given on a five point Likert scale with “not applicable” as a possible sixth response option. For the evaluation, we used the corrected answer key [16]. Scores lie in the number range 0–100, where 0 corresponds to a low and 100 to a high HRQoL measure.

### Statistics

Statistical data analysis was performed with R, version 4.2.3 [34]. NCIQ responses are fundamentally ordinal data. With the exception of the calculation of mean, standard deviation (SD), and effect size (ES), nonparametric methods were used, namely the Wilcoxon matched pairs signed-rank test for location (R-package coin, version 1.4–2, [35]). ES were calculated as Hedges’ g which provides a bias correction for small sample sizes (exact method, R-package effectsize, version 0.8.3 [36]). In the categorization of ES, we follow Cohen’s criteria, where ES > 0.2 correspond to small, ES > 0.5 to medium respectively moderate, and ES > 0.8 to large effect size [37]. Where applicable, ES > 0.5 corresponds to clinically significant effect size [29, 38]. Graphs were prepared with ggplot2, version 3.4.2 [39].

## Results

### Participants’ demographics and clinical data

Demographics and clinical characteristics are presented in Table 1.

**Table 1:** Participants’ demographics and clinical data

**Demographic category**		**Numerical data**
**Gender (male / female)**		9 / 8
**Age at surgical procedure**		63.4 ± 15.8 years (range 18.9 – 87.1 years)
**Age at onset of deafness**		56.2 ± 21.9 years (range 11.9 – 86.7 years)
**Duration of deafness before CI surgical procedure**		7.2 ± 10.3 years (range 0.0 – 36.0 years)
**Categorized duration of deafness pre CI surgical procedure**		
> 1 year		8
< = 1 year		5
= 0		4
**Etiology**		
Acoustic trauma		1
Earlier middle ear surgery		3
Ménière's disease		1
Mumps		1
Otitis media		1
Otosclerosis		1
Sudden hearing loss		5
Unidentified hereditary		1
Unknown		3
**Implanted side (right / left)**		13 / 4
**CI manufacturers (Advanced Bionics / Cochlear / MED-EL)**		3 / 9 / 5
**Hearing status of the contralateral ear**		
Normal		7
Mild hearing loss		1
Moderate hearing loss		6
Profound hearing loss		2
Residual hearing		1
**Duration of CI use within the scope of the study**		1.8 ± 0.6 years (range 0.8 – 2.9 years)

In taking medical history, participants were asked if and when they decided to stop using conventional hearing aids due to lack of benefit. This timepoint was considered the onset of functional deafness and the duration of deafness was defined as the time between this onset and CI surgery. Durations of deafness and etiologies are presented in Table 1.

In all cases, electrode arrays were inserted completely via the round window or extended round window approach. The mean postoperative care duration within the scope of this study was 1.8 years.

### Completeness of questionnaire responses

The percentage of items not answered or answered as not applicable was for BSP 0.6%, for ASP 1.4%, for SP 2.7%, for SE 2.2%, for AL 4.3%, for SI 7.8%, and for the total score across all subdomains 3.2%.

### NCIQ outcomes

**Fig. 1:**
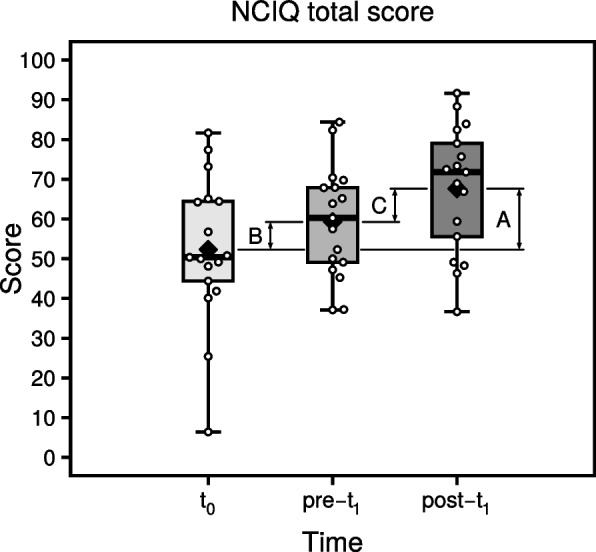
NCIQ total score. The three boxplots represent the scores from the three conditions of answering the questionnaires: preoperative acute (t_0_), preoperative retrospective (pre-t_1_, then-test), and postoperative acute (post-t_1_). Each boxplot shows the total range from minimum to maximum values, the first and third quartile, the median (i.e., second quartile, bold horizontal line) and the mean value (diamond). Three differences, i.e., changes in responses, can be calculated between the three conditions: 1) the post-t_1_ scores minus the t_0_ scores (**A**), 2) the pre-t_1_ (then-test) scores minus the t_0_ scores (**B**), 3) the post-t_1_ scores minus the pre-t_1_ (then-test) scores (**C**)

**Fig. 2:**
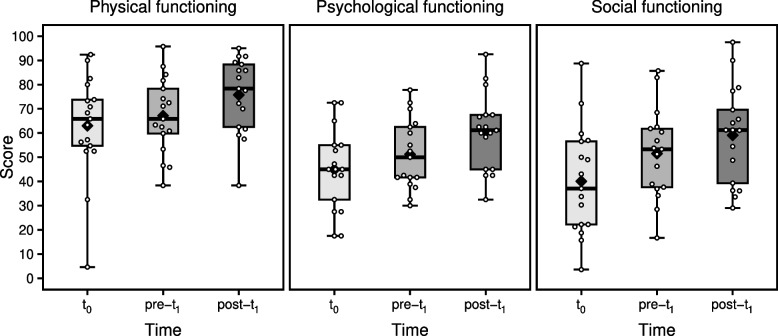
NCIQ general domains: physical, psychological, and social functioning. The boxplots represent the scores from the three conditions of answering the questionnaires: preoperative acute (t_0_), preoperative retrospective (pre-t_1_, then-test), and postoperative acute (post-t_1_). Each boxplot shows the total range from minimum to maximum values, the first and third quartile, the median (i.e., second quartile, bold horizontal line) and the mean value (diamond)

**Fig. 3:**
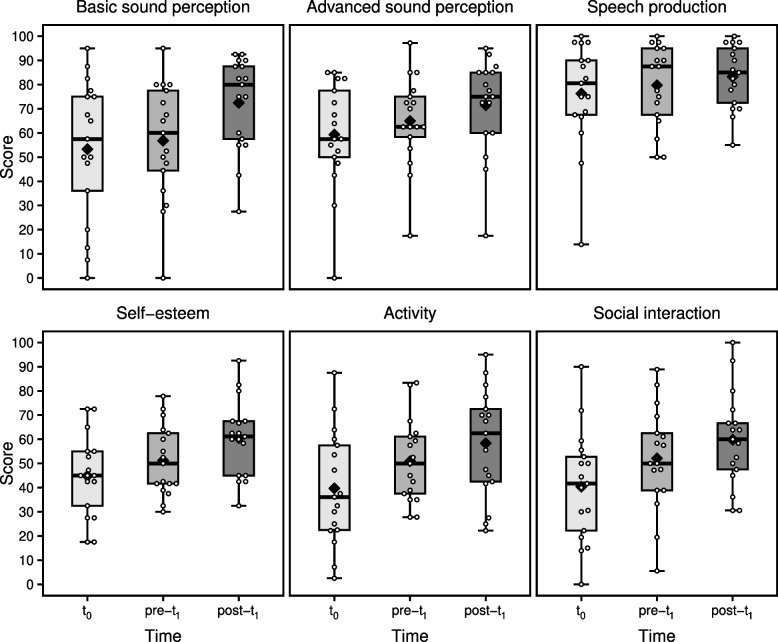
NCIQ subdomains: basic sound perception (BSP), advanced sound perception (ASP), speech production (SP), self-esteem (SE), activity level (AL), and social interaction (SI). The boxplots represent the scores from the three conditions of answering the questionnaires: preoperative acute (t_0_), preoperative retrospective (pre-t_1_, then-test), and postoperative acute (post-t_1_). Each boxplot shows the total range from minimum to maximum values, the first and third quartile, the median (i.e., second quartile, bold horizontal line) and the mean value (diamond)

The NCIQ outcomes are presented in Table 2 and Figs. 1, 2 and 3. Normal distribution of data was tested with the Shapiro–Wilk test. Three of the eighteen tests were not normally distributed: t_0_ SP, post-t_1_ BSP, and ASP. We therefore used nonparametric statistical tests. Participants’ mean total scores were 52.32 (± 18.69) at t_0_, 59.29 (± 14.06) at pre-t_1_, and 67.65 (± 16.02) at post-t_1_.

**Table 2:** NCIQ (total score, general domains, and subdomains) results

	**t**_**0**_				**pre-t**_**1**_** (then-test)**				**post-t**_**1**_			
**Domain**	**Mean**	**SD**	**Median**	**IQR**	**Mean**	**SD**	**Median**	**IQR**	**Mean**	**SD**	**Median**	**IQR**
**Total score**	52.32	18.69	50.36	20.05	59.29	14.06	60.27	18.80	67.65	16.02	71.81	23.43
**Physical**	62.98	21.34	65.83	19.07	67.15	15.78	65.83	18.52	75.75	15.63	78.33	25.83
**Psychological**	44.85	16.72	45.00	22.50	51.21	14.54	50.00	20.83	60.51	15.71	61.11	22.50
**Social**	40.06	22.42	37.08	34.31	51.53	18.41	53.30	24.17	59.07	20.10	61.25	30.28
**Basic sound perception**	53.30	29.27	57.50	38.89	56.80	24.34	60.00	33.06	72.50	19.45	80.00	30.00
**Advanced sound perception**	59.35	22.28	57.50	27.50	64.95	18.54	62.50	16.67	71.47	19.80	75.00	25.00
**Speech production**	76.30	21.90	80.56	22.50	79.71	16.86	87.50	27.50	83.27	13.11	85.00	22.50
**Self esteem**	44.85	16.72	45.00	22.50	51.21	14.54	50.00	20.83	60.51	15.71	61.11	22.50
**Activity**	39.72	23.51	36.11	35.00	50.93	17.12	50.00	23.61	58.35	22.25	62.50	30.00
**Social interaction**	40.40	22.85	41.67	30.56	52.12	21.43	50.00	23.61	59.79	19.64	60.00	19.17

Mean, standard deviation (SD), median, inter-quartile range (IQR) for t_0_, pre-t_1_ (then-test), and post-t_1_

Participants’ physical scores were 62.98 (± 21.34) at t_0_, 67.15 (± 15.78) at pre-t_1_, and 75.75 (± 15.63) at post-t_1_.

The psychological score was 44.85 (± 16.72) at t_0_, 51.21 (± 14.54) at pre-t_1_, and 60.51 (± 15.71) at post-t_1_.

The participants’ social scores were 40.06 (± 22.42) at t_0_, 51.53 (± 18.41) at pre-t_1_, and 59.07 (± 20.10) at post-t_1._.

### Observed change, response shift and then-test change

We calculated three differences (i.e., changes in responses) between the three conditions: 1) The post-t_1_ scores subtracted by the t_0_ scores (A, observed change); 2) the pre-t_1_ (then-test) scores subtracted by the t_0_ scores (B, response shift); and lastly 3) the post-t_1_ scores subtracted by the pre-t_1_ (then-test) scores (C, then-test change). These three differences are shown graphically in Fig. 1 for the total score and can be calculated on both an individual level and a group level.

The time effect is the change in information and behavior over time.

A is called the observed change and includes also the time effect. The A results are statistically significant in all scores except in SP (Table 3).

**Table 3:** NCIQ (total score, general domains, and subdomains) observed changes, response shifts and then-test changes

	**Observed change**				**Response shift**				**Then-test change**			
	**A**				**B**				**C**			
	**post-t**_**1**_**—t**_**0**_				**pre-t**_**1**_**—t**_**0**_				**post-t**_**1**_**—pre-t**_**1**_			
**Domain**	**magn**	**p**	**symb**	**ES**	**magn**	**p**	**symb**	**ES**	**magn**	**p**	**symb**	**ES**
**Total score**	15.33	0.0002	***	1.09	6.97	0.0395	*	0.52	8.36	0.0067	**	0.67
**Physical**	12.76	0.0052	**	0.83	4.17	0.2052		0.26	8.60	0.0130	*	0.55
**Psychological**	15.65	0.0007	***	1.13	6.36	0.0261	*	0.60	9.30	0.0026	**	0.93
**Social**	19.01	0.0005	***	0.94	11.47	0.0395	*	0.63	7.54	0.0395	*	0.51
**Basic sound perception**	19.20	0.0041	**	0.79	3.50	0.4917		0.15	15.70	0.0059	**	0.61
**Advanced sound perception**	12.12	0.0120	*	0.70	5.60	0.0896		0.36	6.52	0.2568		0.35
**Speech production**	6.97	0.2083		0.37	3.41	0.5694		0.19	3.56	0.1697		0.30
**Self esteem**	15.65	0.0007	***	1.13	6.36	0.0261	*	0.60	9.30	0.0026	**	0.93
**Activity**	18.63	0.0033	**	0.79	11.21	0.0174	*	0.61	7.42	0.1268		0.43
**Social interaction**	19.39	0.0042	**	0.92	11.72	0.0553		0.59	7.66	0.0736		0.48

Magnitude of change, Wilcoxon matched-pairs signed rank test *p*-value and its symbolic representation, effect size

B represents the RS. In all cases the sign of the RS was positive. The B results were statistically significant (*p* < 0.05) for total score, psychological domain, social domain, SE, and AL, but not in physical domain, BSP, ASP, SP or SI (Table 3).

C is the then-test change but is in the literature also referred to as true change because the two testing situations start with the same internal standard of measurement. The C results where statistically significant in all scores except SP, AL, SI, and ASP (Table 3).

### Effect size

The ES results are presented in Table 3. The ES of the observed change (A) were greater than 0.8 in the total score, and the three general domain scores, i.e., large and clinically significant. All ES of subdomains except SP were at least moderate and thus clinically significant.

The ES of the response shift (B) were moderate (ES > 0.5) in total score, psychological, social general scores, SE, AL and SI subdomain scores. The ES of the response shifts (B) were small (ES > 0.2) in the physical general domain and ASP subdomain score.

The ES of the then-test change (C) were large for the psychological general domain, moderate for the total score and physical and social general domains, and thus clinically significant. ES of the subdomains were moderate for BSP and small for ASP, SP, AL, and SI.

## Discussion

To our knowledge, this is the first study that demonstrates the existence of a RS in CI users. We found a positive RS for all areas, reaching statistical significance in the total score, in the general domains psychological and social and in the subdomains SE and AL.

To detect RS, we used the then-test method, which is often criticized for being susceptible to recall bias [29, 40] and may contain noise [41]. The deactivation of the CI helps participants to recall their preoperative state and thus minimized bias and noise. However, this allows the possibility that CI users do not in fact think back, but evaluate their temporary present state under the condition with a deactivated CI. In the cases where preoperatively any residual hearing was present, this may be preserved despite the implantation, however, is lost in the majority of cases [42]. Nevertheless, we found that the pre-t_1_ (then-test) results were equal or better than the t_0_ preoperative results. This leads us to the conclusion that the preoperative state actually dominated their then-test responses.

A RS in HRQoL has been investigated in numerous categories of diseases, from mild to life-threatening, including chronic and terminal conditions [26]. Cochlear implantation requires surgery and rehabilitation, after which CI recipients oftentimes can go back to their previous lives before hearing loss.

Korfage et al. [27, 43] found evidence for a RS in men with prostate cancer. The studies indicated that the RS was primarily induced by the cancer diagnosis per se and was larger in size than that induced by the treatment. However, the ES were small to negligible. In people with functional deafness, the diagnosis does not provide them with fundamentally new information due to the participants’ apparent knowledge that their hearing had reduced. Thus, a RS induced by the diagnosis hearing loss cannot occur. In comparison to the prostate cancer studies, we found that the ES of the treatment RS (B) was larger within our study. The direction of the RS (then minus pre) in the prostate cancer patients study was negative, perhaps due to a deterioration in health, however the authors stated it as interpretable for prostate cancer patients [27].

Bernhard et al. [44] evaluated the then-test method by investigating ten QoL indicators via visual analog scales (VAS) in two consecutive treatments in colon cancer patients, first a radical resection surgery and second a post-operative adjuvant chemotherapy composed of three randomized treatment arms, one of them observation. The resection surgery generally resulted in a worsening (i.e., negative observed change) and negative RS in the physical indicators “physical well-being”, “tiredness”, and “functional performance”, while causing improvement (i.e., positive observed change) and no significant RS in the psychological indicators “mood”, “perceived adjustment”, and “anxiety”. The adjuvant treatment arms showed no (6 indicators), positive (3 indicators) or inconsistent between treatments (2 indicators) observed change, generally smaller than the changes around resection surgery. As a result of the negative RS, the then-test change was positive in 9 out of the 10 indicators. However, selectivity between treatment study arms did not improve.

In contrast to the physical indicators regarding surgery treatment in Bernhard et al. [44], in our study the observed changes were significantly positive, and the RS effect sizes were positive, though small. This may be due to the physical indicators in Bernhard et al. [44] addressing physical health state shortly after surgery, whilst our study addressed functional hearing performance after the consolidation phase in hearing rehabilitation. However, both studies showed a RS that followed the direction of the observed change. Psychological indicators yielded improvement in the observed change in both studies, however the RS was significantly positive with medium ES in our study and not significant in the study of Bernhard et al. [44].

Thus, when considering the observations of Bernhard et al. [44], utilizing the then-test method for selectivity between treatments appears questionable. The CI is currently the only existing treatment for rehabilitation of functional deafness, so the question doesn't arise, but that may change in the future.

People with chronic illness type I diabetics with unsuccessful pancreas and kidney transplant procedures, retrospectively overestimate their pre-transplant QoL [45]. Adang et al. [32] demonstrated that type I diabetics with successful transplant procedures underestimate their pre-transplant QoL. These observations suggest that the RS could be outcome dependent. Similar to our results, Adang et al. [32] found significant positive observed change, sustained over an extended period of time after the surgical intervention. The RS was negative though, analogous to other life-threatening conditions, such as cancer [27, 44].

Joore et al. [33] demonstrated that a RS in QoL was present in hearing impaired adults after hearing aid fitting. The RS was primarily seen in the hearing related QoL dimensions and not the generic dimensions that were used as control. Although it could be assumed that hearing aids and CIs are comparable regarding hearing improvement, we in contrast primarily observed a RS in the psychological and social domains. Moreover, the RS observed by Joore et al. [33] were negative with hearing aids, whereas we found positive RS after CI provision.

Joore et al. [33] mention the reluctance of many older persons to accept the necessity of a hearing aid. Their pre-treatment responses could therefore be more positive (since they are not realistic) than their post-treatment responses (which should better match reality), thus resulting in a negative RS. A substantial difference between the groups is the severity of the impairment: while many people can cope with the hearing handicap caused by mild to moderate hearing loss (which can still be improved by conventional hearing aids), unaided functional deafness is more difficult to compensate and/or ignore. At the time of the t_0_ pre-test, the final decision for CI surgery had already been made and a still unrealistically positive assessment of one’s hearing status is unlikely. In comparison, the prescription and prospective use of a hearing aid for an extended time may appear much less severe and final and thus still leave room for handicap denial.

The mean age of participants in the Joore et al. study [33] was 67 years (± 12), while in our cohort the mean age was 63.4 years (± 15.8), rendering the age too similar to explain the diverging differences in the RS. A possible difference could be that the participants within our study were advised to temporarily deactivate their CI to help them recall the preoperative state.

In our study, the RS was positive, likely because the participants raised their standards as a result of hearing improvement and possibly also hope for further improvement with extended use.

According to the meta-analysis conducted by Schwartz et al., the size and the direction of the RS vary considerably in the literature [26]. In this meta-analysis the authors discuss, that in the case two studies investigating similar circumstances reveal different RS directions, then the sign of the RS is relevant and should lead to questioning the validity of the findings [26]. In our study we found that the largest RS had occurred within the social domains and the largest ES in the social and psychological domains. This is in accordance with the hypothesis of Schwartz & Sprangers [24] that a RS is more likely to occur within subjective rather than objective domains.

Regarding the small effect found within the SP subdomain, this is probably attributable to the participants having acquired deafness postlingually and therefore having nearly normal speech production to begin with. To find an effect here, a study would likely need to include participants with bilateral peri- or prelingual deafness. The effect of CI provision on HRQoL is important and can be used to help give CI candidates realistic expectations on postoperative results [9].

As shown by many other studies, where the post-test minus the pre-test was used, CI provision leads to a statistically significant improvement in the HRQoL in all subdomains of the NCIQ [8, 9, 15, 19]. These conclusions are in accordance with our results of the observed change in most scores.

Our null hypothesis that the participants’ preoperative t_0_ scores and the pre-t_1_ (then-test) scores do not differ had to be rejected. This study is the first one to compare the pre-test and the then-test in the same study population in CI users.

The present study is not without limitations. Future studies would benefit from including more participants than the 17 in the present study, which is a small cohort compared to other studies which used the NCIQ [46–48]. As proposed by Sébille et al. [49], different methods should be applied to the same data set to see how they compare with respect to detecting the same type of RS.

## Conclusions

There is a statistically significant RS in the HRQoL of CI users, predominantly in the psychological and social domains. The ES of the RS is moderate and thus clinically significant for these domains and small for the physical domain. The sign of the RS is positive, i.e., it follows the direction of the observed change. In all domains except SP, the observed changes are positive and statistically significant, and their ES are large and thus clinically significant. In the provision of CI, clinicians can expect large positive HRQoL changes in the CI users’ own assessment. In retrospective judgment after extended CI experience, CI users may overestimate their HRQoL in comparison to their preoperative view, especially in the psychological and social domains. For further clinical implications and for the investigation of influencing factors larger studies should be conducted.

## Data Availability

The datasets generated and analyzed during the current study are not publicly available due to the policies of the clinic where the study was conducted, but are available from the corresponding author on reasonable request.

## Abbreviations

AL: Activity level (NCIQ subscore)
ASP: Advanced sound perception (NCIQ subscore)
BSP: Basic sound perception (NCIQ subscore)
CI: Cochlear implant
ES: Effect size
HRQoL: Health related quality of life
IQR: Inter quartile range
magn.: Magnitude
NCIQ: Nijmegen cochlear implant questionnaire
QoL: Quality of life
RS: Response shift
SD: Standard deviation
SE: Self esteem (NCIQ subscore)
SI: Social interaction (NCIQ subscore)
SP: Speech production (NCIQ subscore)
symb.: Symbolic representation
VAS: Visual analog scale
